# Copper Homeostasis in Mammals, with Emphasis on Secretion and Excretion. A Review

**DOI:** 10.3390/ijms21144932

**Published:** 2020-07-13

**Authors:** Maria C. Linder

**Affiliations:** Department of Chemistry and Biochemistry, California State University, Fullerton, CA 91831, USA; mlinder@fullerton.edu

**Keywords:** copper, secretion, excretion, liver, kidney, bile, saliva, gastric fluid, pancreatic fluid, urine, ceruloplasmin, ATP7A, ATP7B, small copper carriers

## Abstract

One of the hallmarks of Cu metabolism in mammals is that tissue and fluid levels are normally maintained within a very narrow range of concentrations. This results from the ability of the organism to respond to variations in intake from food and drink by balancing excretion, which occurs mainly via the bile and feces. Although this sounds straightforward and we have already learned a great deal about aspects of this process, the balance between overall intake and excretion occurs over a high background of Cu recycling, which has generally been ignored. In fact, most of the Cu absorbed from the GI tract actually comes from digestive fluids and is constantly “re-used”. A great deal more recycling of Cu probably occurs in the interior, between cells of individual tissues and the fluid of the blood and interstitium. This review presents what is known that is pertinent to understanding these complexities of mammalian Cu homeostasis and indicates where further studies are needed.

## 1. Introduction

**Figure 1:**
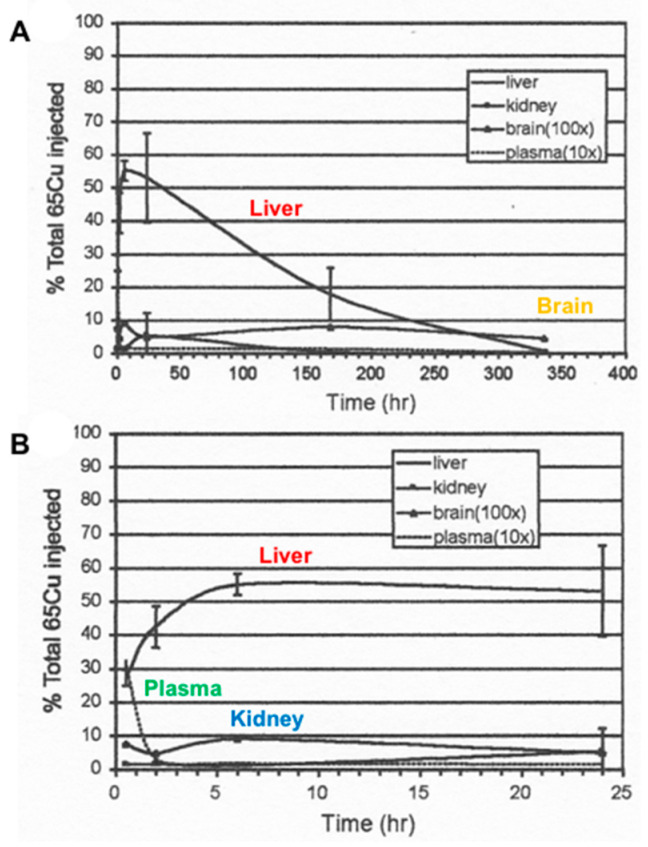
Time course of loss (**A**) and incorporation (**B**) of excess extrinsic ^65^Cu from mouse organs after the injection of this stable isotope. Mice were preloaded with 3 times more Cu than the total estimated to be in their bodies, over 14 h, after which they were sacrificed at various times, as indicated, and the percent dose was determined. Data for plasma and brain were multiplied 10× and 100×, respectively, to make them visible. Annotated, and reprinted with permission from Springer, Biometals, Copper binding components of blood plasma and organs and their response to influx of.

The term “homeostasis”, or the maintenance of constancy within an organism, aptly applies to Cu in mammals. The constancy of tissue and fluid concentrations of this trace metal is truly remarkable, and not only so within the same species but across almost all mammals (with the exception of the dog and perhaps some others as yet not identified). Liver concentrations are usually about 5 µg/g; heart concentrations are also about 5 µg/g; brain concentrations are 4–5 µg/g; muscle concentrations are ~1 µg/g, and so on [1]. Kidney concentrations are more variable, at around 4–12 µg/g. It is also remarkable how robustly mammals can respond to receiving an excess of Cu. In the case of mice, for example, loaded with three times their normal total amounts of body Cu, concentrations of this element in the organs and fluids virtually returned to normal after about 2 weeks [2] (Figure 1). It is well understood that the liver plays a major role in remediating this and other kinds of Cu overload by transferring excess Cu to the bile, which then returns it to the gut for fecal excretion. Indeed, it is generally assumed that this is the way mammalian Cu homeostasis is regulated. This idea is based mainly on the findings from the most well-studied disease of Cu overload (Wilson disease), first identified in humans and involving the “Cu pump” ATP7B [3,4], and on findings for Cu overload in dogs involving other proteins required for putting Cu into bile, namely COMMD1 [5,6] and ABCA12 [7]. Moreover, it has long been known that very little copper is excreted through the urine (on the order of 50 µg/day). What has generally been ignored, however, is that a considerable amount of Cu is secreted into the gastrointestinal tract daily, beyond that contributed by bile and the diet. This Cu (from saliva, gastric, pancreatic and intestinal juices) is of course largely recycled (meaning re-absorbed, re-distributed and re-secreted). How that all happens, and its implications for understanding Cu homeostasis, has not particularly been considered. This review addresses current knowledge about these and other aspects of Cu metabolism that affect homeostasis, and highlights where additional studies are needed to better understand this process.

## 2. Secretion and Recycling of Cu in Gastrointestinal Fluids

The average 70 kg human adult has a total of about 110 mg of Cu in the body [8]. Dietary intake is about 1 mg per day [9]. Along with food and drink, many liters of secreted fluids enter the digestive tract daily, averaging roughly 1–2 L for saliva, with 2.5 L of gastric fluid, 1.5 L of pancreatic fluid, 0.5–0.75 L of bile, and unknown volumes of intestinal fluids, for the average 70 kg adult [1]. Each of these fluids contain Cu (Table 1). Multiplying concentration by volume indicates that more Cu comes into the GI tract through these secretions than comes in from outside (Table 1). About one milligram comes from saliva, the stomach and pancreas; 0.6–6 mg are added by the bile, totaling from 1.6 to 7 mg per day not coming from food and drink. Balance studies as well as those using radioactive or stable Cu isotopes indicate that in humans, 50–80 percent of Cu in the GI tract is absorbed [10]. Absorption occurs primarily (and probably exclusively) in the small intestine. This means that much more Cu is entering enterocytes than that in the diet. Since homeostasis is the balance between intake and output, this needs to be taken into account, and what happens to the non-dietary portion needs to be included.

**Figure 2:**
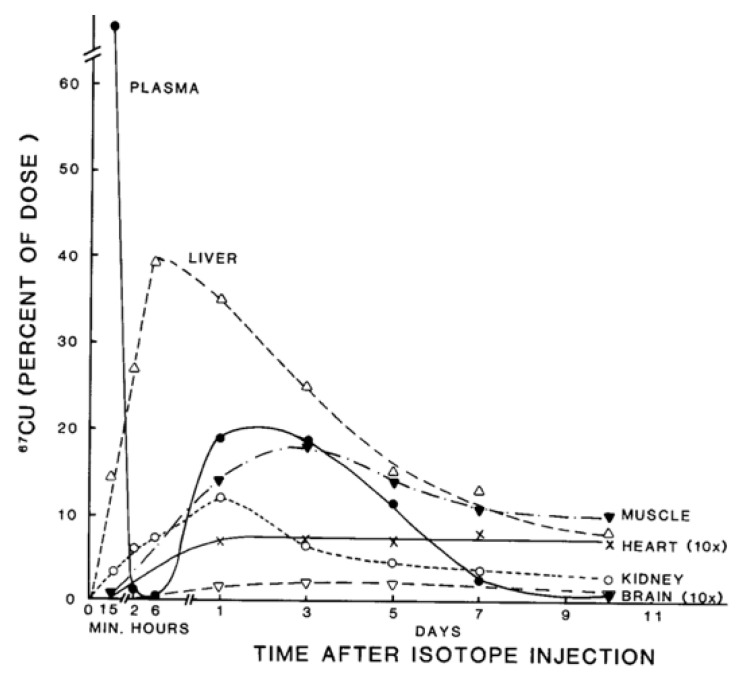
Distribution of ^67^Cu in rat organs and blood plasma at various times after tracer administration. Data for each time point are mean values for mostly 5–7 rats, given as % of ^67^Cu dose. Data for brain and heart have been multiplied 10× to make them more visible. Reprinted with permission from reference Weiss, K.C.; et al. [14].

Almost all of the Cu absorbed by the small intestine enters the hepatic portal vein and thus the liver. Tracer studies in humans and rodents indicated that most of it initially enters the cells of this organ [1,10,14,15] (see example in Figure 2). Assuming that the tracer is assimilated in the same way as other Cu ions entering the gut, enterocytes and blood, this means that most of the recycled Cu (coming from GI secretions) also initially goes into liver cells. In hepatocytes, the three main things we know happen to the incoming copper are (a) entry of some of it into endogenous Cu-dependent proteins; (b) incorporation of a significant portion into ceruloplasmin, for secretion into the blood; and (c) incorporation of excess into the bile to maintain homeostasis through excretion. All of this results in a net daily excretion of about 1 mg Cu in the feces, balancing the ~1 mg Cu taken in through food and drink. 

Clearly, a great deal of Cu enters the GI tract in secretions and is recycled. However, not all secretions are recycled equally. Existing data indicate that the Cu in bile is not only more tightly bound to biliary components, but is less re-absorbable than that in other GI fluids (Table 2). The amounts of Cu secreted into the bile are also highly variable among human individuals (Table 1). These characteristics of bile are consistent with a role in maintaining the constancy of Cu levels within the organism through the adjustment of the amounts and forms of Cu secreted into bile that contribute to fecal excretion. The regulation of Cu homeostasis is thus primarily through biliary excretion rather than changes in the degree of absorption, so much Cu is re-absorbed and recycled from the GI tract, and excretion adapts to how much needs to be retained. 

## 3. Biliary Cu Excretion

**Figure 3:**
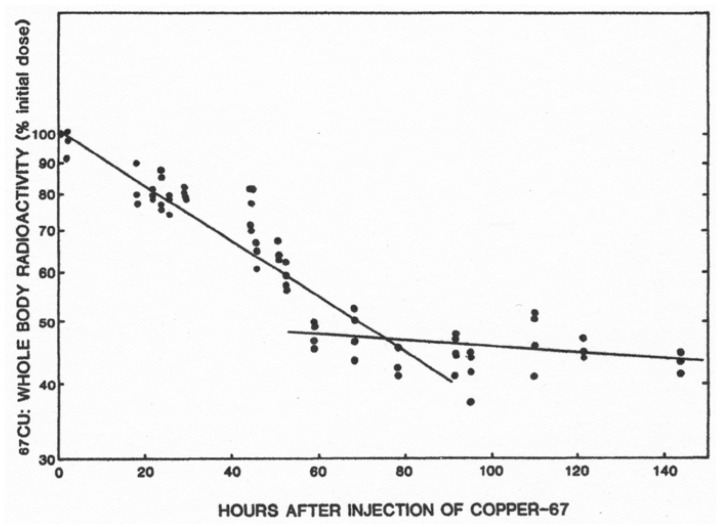
Effect of bile duct ligation on turnover of whole body Cu in rats. Tabulated data on rates of ^67^Cu loss in rats where the common bile duct was and was not (sham) ligated prior to ^67^Cu administration. Reprinted with permission from Linder, M.C. et al. [2].

The importance of the biliary route for Cu excretion is clearly demonstrated by what happens when this route is compromised, notably when proteins associated with mediating steps in the process of ferrying Cu into bile are defective, and/or if the common bile duct is ligated, as in experimental animals. Wilson disease, in which a Cu “pump” (ATP7B) in hepatocytes is dysfunctional, results in accumulations of large amounts of Cu in the liver that eventually lead to hepatic Cu overload as well as excess accumulation in some other tissues, such as the brain [3,4,16]. This is lethal if untreated. As shown in rats, ligating the bile duct results in a marked decrease in the rate of loss of whole-body Cu. Figure 3 shows the loss in normal rats preloaded with ^67^Cu and monitored for 7 days [2]. Rates of loss of a faster and slower component or route are evident, with the half-life of the faster one being 54 h (Table 3). This rate of Cu loss was cut in half by ligation (t_1/2_ going to 117 h). Biliary Cu secretion is very responsive to Cu treatment. Long-Evans Agouti rats, for example, increased biliary Cu concentrations 2–3 fold within minutes of receiving an injection of Cu-his [17]; 

Biliary Cu levels of Wistar rats rose eight-fold over 180 min after the start of continuous intravenous infusion of CuSO_4_ (318 ng/g body weight/h) [18]. As already mentioned, Cu concentrations in bile (mg/L) are also quite variable, with standard deviations for mean values of human bile being on the order of 50% (4 + 2 [1]; 3.6 + 1.7 [19]) (Table 1). Presumably, this reflects adjustments made by hepatocytes in response to different levels of Cu being absorbed by the intestine and entering the liver.

**Figure 4:**
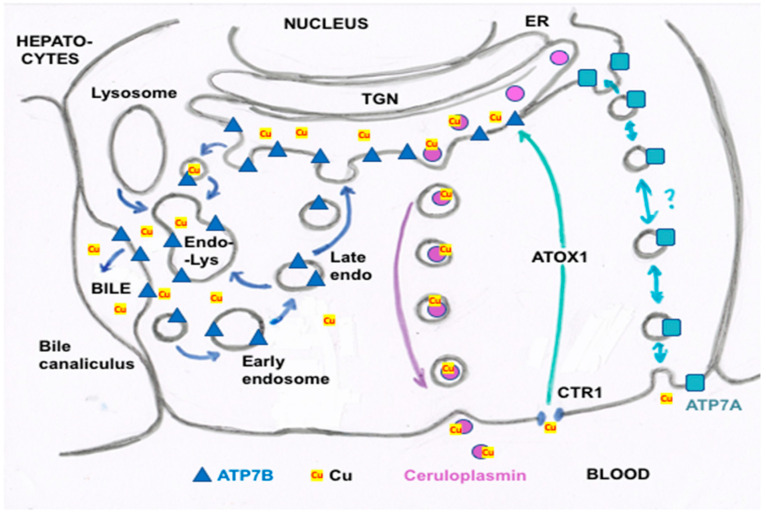
Summary of proposed steps involved in the movement of cu into bile and secreted by hepatocytes. Based mainly on the data, reviews and figures provided by Polishchuk and Polishchuk [26] and Stewart et al. [27]. Cu ions enter hepatocytes from plasma proteins via copper transporter 1 (CTR1) and at least one other as yet unidentified transporter, and are carried via the chaperone ATOX1 to ATP7B (blue triangles) in the transGolgi network (TGN). In the lumen of the TGN, Cu will be incorporated into apoceruloplasmin (apoCp) (violet circles), forming holoCp, and be released into the blood plasma by exocytosis (center right of the figure) across the “basolateral” hepatocyte membrane. In the absence of excessive Cu not otherwise incorporated into Cp and endogenous Cu-dependent proteins and mitochondria, TGN Cu in the lumen will be exported to the bile canaliculi that are formed between hepatocyte apical membranes, leading to the gall bladder and bile duct. This happens through the budding of vesicles from the TGN that contain Cu and may also contain ATP7B. These fuse with late endosomes also containing ATP7B as well as lysosomes (to form the endo-lysosomal compartment), which then fuses with the apical hepatocyte membrane at a canaliculus (left side of figure). This releases Cu that was present in the vesicular bodies and also can lead to ATP7B being part of the apical membrane. The latter is particularly important in the presence of excess Cu, which can then flow from ATOX1 directly to ATP7B that pumps it into the bile canaliculi. Small amounts of the Cu efflux “pump”, ATP7A (blue squares), are also expressed in hepatocytes (far right of the figure), and may also contribute to Cu that exits hepatocytes and enters the blood plasma, to bind to proteins like albumin in the exchangeable plasma Cu pool. Based on what is known in other cells, the much less abundant ATP7A in hepatocytes would either transfer some Cu in the TGN into secretory vesicles (for exocytosis), and/or traffic it in vesicles to the basolateral membrane to pump Cu in the cytosol (on ATOX1) into the blood plasma, following which ATP7A would recycle back to the TGN.

We are continuing to learn about the mechanisms by which Cu entering the liver and hepatocytes is forwarded to the bile, particularly with the help of ATP7B (Figure 4). ATP7B functions not only as a means of ridding the body of excess Cu entering hepatocytes but to supply the metal ion to Cu-dependent proteins that are produced and secreted into the extracellular fluid and blood by hepatocytes and some other cells [20]. In certain cells, such as enterocytes and kidney epithelia, it may also function to sequester/store Cu in vesicles [21,22]. Hepatocyte ATP7B sits in the membrane of the trans Golgi network (TGN), pumping Cu from the cytosol into the TGN lumen (Figure 4). Here it encounters apo-ceruloplasmin that has been synthesized in the rough endoplasmic reticulum (ER) and glycosylated in the smooth ER, which then receives the Cu in the TGN. From there, holo-ceruloplasmin (containing Cu) proceeds to the blood (with other secreted proteins) via exocytosis across the “basolateral” portions of the hepatocyte membrane (Figure 4). This is most probably a constant process, with rates of ceruloplasmin synthesis being fairly constant in the liver under normal circumstances [23]. Cu not needed for incorporation into ceruloplasmin and other proteins (including endogenous ones like SOD1 and cytochrome c oxidase) appears to be trafficked (with ATP7B) from the TGN into late endosomes/lysosomes (the endolysosomal compartment) [24]. More importantly, when large amounts of excess Cu enter hepatocytes, endolysosomal vesicles with ATP7B traffic it towards the bile canalicular (“apical”) portions of the hepatocyte membrane, and appear to fuse with it (Figure 4, left side). This would release the Cu that is inside the vesicles into bile canaliculi, while also bringing ATP7B itself to the apical membrane to directly pump excess Cu from cytosolic ATOX1 into the developing bile. The hepatocyte apical-targeting sequence, FAFDNVGYE, is near the N-terminus of ATP7B.) These processes are summarized in Figure 4. (For further details, please see reviews by Polishchuk and Lutsenko [25], and Polishchuk and Polishchuk [26], as well as Stewart et al. [27].)

When levels of excess Cu in the hepatocyte subside, ATP7B recycles back into the TGN, via a clathrin-dependent endocytic process (Figure 4). This depends on a leucine triad located in the C-terminal portion of the ATP7B that is recognized by adaptins [27]. Another protein (COMMD1, formerly MURR1) is also necessary for biliary Cu excretion, as first demonstrated in Bedlington terriers suffering from liver Cu toxicosis [5,28]. COMMD1 was the first member of what is now recognized as a family of ten proteins involved in endosomal sorting, and also plays a role in endosomal recycling [27]. As a complex with two other proteins (CCDC22-CCDC93), it binds to ATP7B in early endosomes, and somehow assists its return to the TGN [26] (Figure 4). In its various locations (TGN, vesicles, plasma membrane), Cu is provided to ATP7B by the cytosolic Cu chaperone, ATOX1 (HAH1). A third protein (ABCA12) involved in ferrying Cu to the bile has also been identified in Bedlington terriers [7]. Although its exact role is still unclear, it may be functionally similar to ATP7B. 

Biliary excretion probably varies from minute to minute, as ATP7B and other proteins respond to cytosolic Cu concentrations in hepatocytes by staying in the TGN or moving to apical portions of the plasma membrane, as previously explained (Figure 4). In general, it is thought that trafficking of existing ATP7B rather than changes in levels of this protein (or that of COMMD1) is responsible for dealing with the day-today routine of sending the right amounts of Cu out of the liver into the bile to maintain homeostasis [28,29]. Expression of ATP7B is probably quite stable under normal physiological conditions, as also suggested by measurements of mRNA in adult rat liver [23], and relatively little has been done to establish what factors might alter expression and/or rates of synthesis and degradation of ATP7B and COMMD1 in hepatocytes or other cells. However, it has been known for some time that the promoter/enhancer regions of the ATP7B gene contain several metal-responsive elements (MREs) [30], to which metal transcription factors (MTFs) could bind. Several reports indicated that large doses of Cu evoke increased ATP7B expression in the livers of very young animals, such as rat pups [31] and piglets [32], as well as in seabream [33]. The recent work of Stalke et al. [34], which employed a reporter gene construct in HepG2 cells, provided evidence that enhanced ATP7B expression induced by Cu involves binding of MTF1 to the “e” MRE in the gene regulatory region. In young fish (guppies), Cu treatment was found to increase Atp7b (and Ctr1) mRNA expression in the gut and gills but not in the liver [35]. In cultured human skin fibroblasts, small and inconsistent effects of adding Cu (concentrations unknown) or Cu chelators on mRNA expression were observed for ATP7B, but there was a more clear-cut positive effect when adding the Fe(II)-chelator, ferrozine, to cause iron deficiency [36]. For suckling and adult rat duodenum, there was a report that protein expression of the ATP7B homolog, ATP7**A**, was enhanced by iron deficiency, and induced its localization to the brush border/apical rather than basolateral membrane, the latter of which occurs in response to Cu [37]. However, a multifaceted study of the response of Atp7a to Cu treatment and deficiency showed that, in cultured intestinal (IEC-6) cells, Cu induced no immediate changes in mRNA (over 3 h) but markedly increased protein expression by decreasing the degradation of Atp7a, relative to Cu deficiency. Longer term (12 h) Cu treatment of cultured 12 day old mouse liver cells showed large increases in Atp7a over that in Cu deficiency, with the same being true in human vascular epithelial cells (HUVEC). Seven day old mice treated daily (for three days) with Cu only demonstrated an increased liver Atp7a protein expression at very high doses, doses that also increased the blood serum levels of Cu (suggesting toxicity), and there were no effects on levels of Atp7b in the liver [38]. Thus, it seems that in certain conditions, and particularly in the organs of the very young, Cu and perhaps also Fe have roles in regulating expression of these P-type Cu ATPases. 

As concerns COMMD1, its overexpression in HEK 293 cells was shown to enhance degradation of stably expressed ATP7B in wild type as well as Wilson disease-mutated cells [39]. The same was then shown for ATP7A [40]. Indeed, both of these ATPases bind COMMD1, as well as clusterin (a chaperone protein that attempts to restore structure), as shown by co-immunoprecipitation [41]. Interestingly, stably (but not transiently) overexpressing either of these proteins in HEK cells resulted in enhanced degradation of both ATPases, but by different apparent mechanisms. High levels of COMMD1 resulted in less ATP7A and B protein that was prevented by the proteasomal inhibitor, MG132, while the effects of high levels of clusterin were prevented by the lysosomal inhibitors, ammonium chloride and leupeptin. Degradation of oxidized and mutated ATP7B and A was also facilitated by clusterin [40,41]. Whether large increases in levels of COMMD1 and clusterin naturally occur, however, is unclear, and whether these two binding proteins control the normal levels of the ATPases still needs to be established, but is a possibility. 

## 4. Forms of Cu in Bile and Their Origins

What we have described so far with regard to how Cu enters the bile makes the assumption that it comes from that “pumped” by ATP7B from the cytosolic Cu chaperone, ATOX1 [42]. Both ATOX1 and ATP7B bind Cu^1+^ via similar CXXC motifs; and so it is assumed that Cu^1+^ ions are released from the ATP7B either directly into the bile canaliculi, or into the TGN lumenal fluid which enters vesicles destined for biliary exocytosis. Some may also accompany ceruloplasmin into the blood in exocytic vesicles. Cu ions tend to bind to proteins and peptides as well as other ligands, such as glutathione. Therefore, they are likely to bind to a number of components of the developing biliary fluid. The binding may be tight or loose or in between. It may or may not be for the specific purpose of producing a component that is not digestible or reabsorbable in the gut, or has some other purpose. 

Most of the available information on the forms of Cu present in bile comes from studies done in the 1960s to the 1980s, with very few following up in recent decades. The early studies (summarized from Linder [1]) indicated the following about the Cu binding components in bile:

(a) The nature of the components varies in terms of how easily the attached Cu can be dialyzed away and how easily it is re-absorbed by the GI tract, as shown for example in rats injected with 100 µg of ^64^Cu, measuring biliary ^64^Cu radioactivity over time, dialyzing the bile (into 0.9% NaCl), and administering portions into new rats by intrapyloric intubation, to measure rates of intestinal uptake [1]. Table 2 shows that the bile collected early on (zero to 4h after ^64^Cu administration) had half of its Cu loosely bound (and released by dialysis); but dialyzability decreased substantially over time and was accompanied by greater susceptibility to TCA precipitation (a characteristic of proteins). This suggests that a portion of the Cu in bile becomes more tightly bound and is associated with proteins. Reabsorption of the dialyzed and undialysed biliary Cu, however, was similar but quite low (10–15%), reinforcing the concept that biliary Cu is more difficult to re-absorb than that in other GI fluids. In another study with human GI fluids (Table 2), gastric and salivary Cu was easily dialyzed away (85–98%) vs. that of gall bladder bile (12%), using 0.9% NaCl/10 mM Tris HCl buffer, pH 8. Additionally, the absorbability of salivary and gastric fluid Cu (administered intraduodenally to rats) was twice that of gall bladder bile, and the same as for Cu^2+^-acetate or histidine.
(b) The nature and size of the biliary Cu components was determined largely by size exclusion chromatography of human and rat bile that had been labeled in vitro or in vivo with ^64^Cu^2+^. It showed two major peaks in Sephadex G50 and G75, one in the void volume and the other at about 5 kDa. Bile acids (detergents like glycocholate, taurodeoxycholate and lecithin) disaggregated the larger component into the smaller one. Traces of an 8 kDa component [43] and another below 5 kDa [44] were also reported. Where tested, elution of these Cu complexes was not abolished or changed by treating with trypsin or other digestive proteases. This suggests they are not polypeptides, and would explain how they survive the gut for excretion in the feces. Alternatively, the proteins/peptides might for some other reason be resistant to degradation by digestive enzymes. The Cu attached to these components was bound very tightly, as it was not removed by strong chelating agents [1]. Other work suggested that one or more of these components may be fragments of ceruloplasmin that are hard to digest by proteases. In one case [45], but not another [46], some of the Cu of bile eluting in the void volume reacted with the antibody against ceruloplasmin. The former is consistent with physiological studies in rats by another group [47], where ceruloplasmin that had been radiolabeled in vitro with ^67^Cu or ^64^Cu was given i.v., and some of the radiolabeled ceruloplasmin-Cu ended up not only in the liver but in the bile. One caveat related to these studies, however, is that it is very difficult to insert Cu into ceruloplasmin in vitro, and it was not ascertained that the radiolabeled Cu added and used in these experiments was in its native state.)
(c) Other kinds of studies also support the idea that ceruloplasmin or its fragments can enter bile. In other rat studies, more of the radioactive Cu associated with ceruloplasmin entered the bile when administered as asialo-ceruloplasmin [48]. Sialic acid is known to be removed from several plasma proteins by hepatic endothelial cells. This has also been demonstrated for ceruloplasmin [48,49]. Desialylation occurred during transcytosis of the hepatic endothelial cells that form a porous layer between the blood and the hepatocytes sheaves, and the asialo-form then entered the hepatocytes via the galactose receptor [48,49]. In line with this, the binding of ceruloplasmin to specific “receptors” on the surface of hepatic endothelial cells but not hepatocytes was demonstrated histologically and through binding studies by Tavassoli et al. [50]. If ceruloplasmin enters hepatocytes via the galactose receptor, it will be transferred into the lysosomal compartment, which is also a source of bile components (Figure 4). These various studies (which have not been followed up) suggested that a portion or fragment of asialo-ceruloplasmin could be the main form of biliary Cu not re-absorbed by the intestine that ends up in the feces.

**Figure 5:**
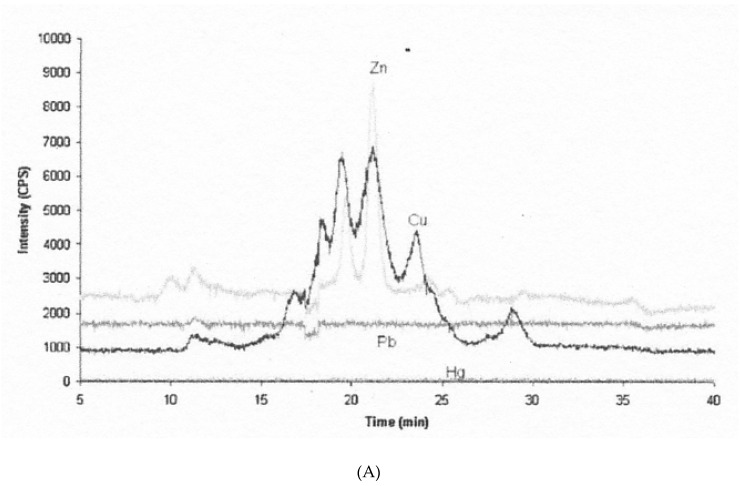

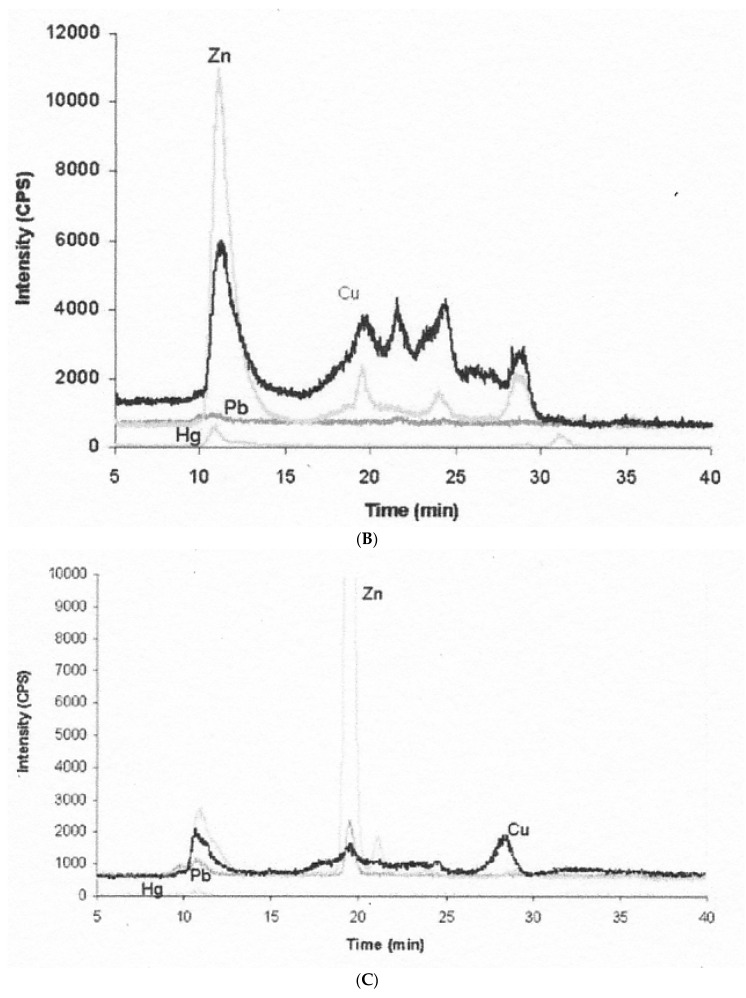
Metallothionein (MT) and other Cu components in fish bile separated in size exclusion chromatography (SEC) coupled to ICP-MS. Bile samples were first heated to remove most other proteins by precipitation, and the supernatants (containing MT) were applied to SEC. Elution of Cu and some other metal ions is shown for bile obtained from fish in uncontaminated waters (**A**), in Cu contaminated waters (**B**), and in waters containing excess Zn (**C**). X-axis is time (from 5 to 40 min); Y-axis is metal isotope intensity for Cu (black), Zn (light grey), as well as Pb (dark grey) and Hg (very pale). Reprinted with permission from Hauser-Davis et al. [51].

The only recent studies on the Cu components of bile appear to be those from fish, carried out in connection with detecting Cu pollution in lakes, and measuring levels of metallothioneins (MTs) in liver and bile. MTs store Cu, Zn and some other heavy metal ions. To determine whether MT was in bile, and that its levels reflected heavy metal pollution (including that of Cu), bile from Cu-loaded and normal Tilapia fish was first treated with heat to precipitate non-MT proteins, and the supernatant was applied to size exclusion HPLC-ICP-MS using Superdex 75 [51]. Whether heat treatment did indeed denature and precipitate most the proteins and/or ceruloplasmin in the bile is unknown. As in the earlier studies summarized in the paragraphs above, there was a major peak in the void volume in the case of fish living in Cu contaminated water (Figure 5B dark line). There also were not just one but 4–5 Cu peaks in the included volume, one of which also contained most of the Zn and so might be MT. The included-volume peaks (but not the void volume peak) were also present in bile from fish living in the reference (low metal contamination) site (Figure 5A), where the Zn content of a peak in the MT position was the most prominent also for Cu. This same peak (at ~ 19 min) was the main one binding Zn in fish from water highly contaminated with Zn (Figure 5C), and the authors indicate that pure MT had eluted there in previous studies using the same column and conditions. The investigators did not confirm that this peak was indeed MT, but they detected MT in whole bile immunologically (by Western blotting). The authors also did not calculate the apparent mass of the peaks they separated from the heat-treated bile samples (the apparent molecular weight of MT is 14 kDa). Thus, whether any of the peaks (and if so which) might correspond to the 5 kDa Cu component seen in mammalian bile in the past is unclear. The investigators also did not attempt to dissociate the Vo peak with detergent, which previously moved Cu from the large to the 5 kDa component. In any event, it seems that at least when fish are Cu-loaded, some Cu enters the bile on MT, and this may also be true for humans and other mammals. In follow-up studies of fish, the authors did find that levels of MT in the liver correlated well with those of MT-Cu found in bile, as would be expected [52]. Therefore, bile appears to contain Cu-binding components that vary in their size and absorbability, and at least small amounts or parts of some Cu proteins (ceruloplasmin and metallothionein) can be there. Obviously, much remains to be learned about the exact nature and relative abundance of these molecules and their roles in Cu excretion versus reabsorption.

## 5. Forms of Cu in Other GI Fluids and Their Origins

As already described, most of the Cu absorbed by the intestine that enters hepatic cells from the portal vein is from fluids that had been secreted into the GI tract. This Cu needs to return to the various cells that produced the secretions to complete the homeostatic loop. We have already described the route taken by Cu to the bile after entry into hepatocytes. Since evidence indicates that normally, most absorbed Cu first goes to the liver, we need to explore what is known about how Cu returns from the liver to those secretory cells and organs that provide saliva, as well as gastric and pancreatic juices.

So far, we have only described our current understanding of the main ways in which Cu leaves hepatic cells, which is by incorporation into ceruloplasmin (in the TGN) for secretion into the blood and interstitial fluid via exocytosis; and transfer across the apical portions of the plasma membrane into the developing bile in the canaliculi. Ceruloplasmin is a major distributor of Cu via the circulation, and delivers it directly to Cu uptake transporters, such as Cu transporter 1 (CTR1), on the surface of many cell types, producing apo-ceruloplasmin in the process [53]. Nevertheless, it seems highly unlikely that even normally (vs. in aceruloplasminemia), ceruloplasmin is the only way in which all this Cu leaves the liver and enters the circulation to return Cu to where it is needed for GI fluid production. Unfortunately, we have almost no information that allows us to estimate how much Cu is released from the liver via ceruloplasmin daily, and thus how much it might be contributing to Cu recycling for GI tract fluids. Rat studies in which the relative half-life of ceruloplasmin protein was calculated (obtained by double labeling with ^3^H- and ^14^C-leucine) only indicated that the turnover rate of ceruloplasmin was 3–4 fold higher than that for plasma proteins as a whole [23]. Clearly, Cu in the bloodstream is also bound to proteins other than ceruloplasmin and potentially also to non-protein molecules. Humans and mice with no ceruloplasmin in the circulation survive fairly well overall, although not without some complications involving both Cu and Fe metabolism [54,55,56,57]. In Wilson disease (with no functional ATP7B to incorporate Cu into ceruloplasmin), there still is Cu in the blood, and subjects lead relatively normal lives as long as they are treated with chelators to remove the excess Cu [3]. So how does the Cu in hepatocytes get back into the circulation from the liver other than on ceruloplasmin, and in what other form(s) is it released? 

One obvious possibility is that Cu entering the TGN through ATP7B from ATOX1 does not just go into ceruloplasmin but also binds to other as yet unknown factors that lead to its secretion, perhaps even accompanying ceruloplasmin in exocytic vesicles that fuse with the (basolateral) plasma membrane (Figure 4). Another obvious possibility is secretion via ATP7A (Figure 4). The homologous Cu “pumps”, ATP7A and ATPB, carry out Cu transport in different ways. While ATP7B is expressed in a limited number of cell types (notably the hepatocyte and mammary epithelial cells) and provides Cu to secreted proteins (such as ceruloplasmin in hepatocytes, and lysyl oxidase in fibroblasts, ATP7**A** is expressed in most cells and mainly releases Cu into the blood not bound to proteins [58,59]. Thus, knocking out ATP7**B** prevents or markedly reduces Cu entering ceruloplasmin or the bile, causing hepatic Cu accumulation, while knocking out ATP7**A** greatly diminishes the transfer of cellular Cu into the blood, the most notable example being the release from enterocytes during intestinal absorption. However, it has become clear that hepatic cells express not only ATP7B but also some ATP7A [38], and this would ostensibly contribute to the transfer of Cu from the liver to the rest of the organism. 

The form in which this Cu emerges after being pumped across membranes by ATP7A and ATP7B is not entirely clear. Delivery of the Cu to these membrane transporters occurs via binding to CXXC motifs as Cu^1+^ on ATOX1 [60], followed by transfer to the same kinds of motifs on ATP7A or B. Thus, Cu appears to enter into the membrane transporters as Cu^1+^ in line with the reducing environment of the cytosol. If, on the other side of the membrane, the Cu released is also Cu^1+^, with ATP7A it would enter the non-reducing (oxidizing) environment of the blood fluid. Based on radiotracer studies, Cu released by ATP7A after intestinal absorption immediately appears on albumin and transcuprein (macroglobulin) in the blood plasma [14], and these proteins bind Cu^2+^ [61,62]. Therefore, the Cu^1+^ would have to be oxidized after release. This could just be induced by oxygen itself coursing through the blood. Alternatively, Cu^1+^ might be oxidized during transit through the transporter. Whether the Cu^1+^ delivered by ATP7B to the TGN lumen also encounters an oxidizing environment is unclear, but is also less likely. Still another possibility has been proposed, namely that both ATP7A and B might be able to transport not only ionic Cu but also metal complexes, such as the anticancer drug cisplatin and its analogs [63,64]. Mediation of uptake of these drugs by cells and their transfer across internal membranes has been demonstrated to involve other Cu transporters, namely CTR1 and CTR2, respectively [65,66]. Uptake of these complexes may, however, not take the same route as that for the Cu ion, and more studies are needed to clarify whether and how metal complexes (as opposed to Cu ions) interact with these various transporters. 

Other as yet unknown ways by which hepatocytes release Cu in some form may also exist. As already indicated, ionic Cu released from hepatocytes will bind to albumin and transcuprein [67] and perhaps also to other proteins and/or peptides that make up the exchangeable Cu pool in the blood plasma. It will travel on these proteins (as well as ceruloplasmin) in the circulation, and, despite binding tightly, be delivered directly to transporters (or transporters complexed with reductases to reduce Cu^2+^) on the surface of cells, as shown in tissue culture with several types of cells [53,68,69]. Except for CTR1, the Cu uptake transporters involved have not as yet been identified [53,68,69]. Cells of the salivary gland, as well as those of the gastric mucosa and pancreas that produce GI secretions, would thus receive the Cu they need from one or more of these carrier proteins, via CTR1 and Cu uptake transporters. 

Once inside the cell, the Cu would find its way to the secretory pathway, and specifically to the TGN, carried on the Cu chaperone ATOX1. In the TGN, the Cu would encounter and be incorporated into the specific Cu components that are secreted in saliva, gastric, pancreatic and other fluids into the GI tract by exocytosis. What is known about the forms of these components, and the most recent information on the amounts of Cu associated with these fluids, is described below. 

Salivary Copper: There are a few articles in the more recent literature reporting concentrations of this metal in whole/crude saliva and sub-fractions, from normal individuals and those with diseases like cancer of the head and neck [70] and oral submucous fibrosis [71], involving groups of 20–60 subjects. The reported total Cu concentrations in normal saliva are in the ballpark but lower than those reported in earlier decades (more like 0.1 vs 0.2 mg/L, or ~ 1.5 µM). Few attempts have been made to identify to what salivary Cu is bound. One group approached this question by adding Cu to saliva samples, and then separating components by size, shape or other means [72]. To pooled saliva from 5 individuals, Cu (as CuSO_4_) was or was not added to final concentrations of 10–40 µg Cu/L, following which samples were examined for changes in what proteins were present in supernatants, after sedimentation (16,000× *g*, 5 min), or in response to 5 kDa ultrafiltration, and/or upon separation in reverse phase HPLC followed by SDS-PAGE. The findings appear to be contradictory. On the one hand, 85% of the inherent Cu was ultrafiltrable from crude saliva not treated with Cu, thus being in small molecules < 5 kDa. Addition of 10 µM ionic Cu further increased that proportion to 95%, perhaps indicating that the excess Cu was still in ionic form, and thus exceeded the Cu binding capacity of the saliva sample. On the other hand, addition of much higher levels (40 µg Cu/L as Cu sulfate) precipitated portions of the main known salivary proteins, implying that the Cu was binding to them. This reduced the specific proteins remaining in the supernatant that were applied to reverse phase HPLC. The most dominant proteins in the saliva that precipitated with the Cu were alpha-amylase (51–54 kDa) and basic proline-rich proteins (PRPs; ~11 kDa [73]). With lower Cu concentrations (5 and 10 µg/L), there was less precipitation, but also an increase in Cu associated with HPLC peaks that contained proteins of 29 and 33 kDa. These proteins were not identified, but the authors concluded they are unlikely to be PRPs. Thus, we really do not know to what Cu is bound in saliva, particularly under normal conditions, but it seems that at lower (more physiological) concentrations, namely 1–2 µM, most of the Cu is associated with a component (or components) of low molecular weight (< 5 kDa).

In line with this, it has been known for some time that saliva contains a group of basic, histidine-rich peptides, histatins, that can bind with copper and are 3–4 kDa in mass [74,75,76]. They contain N-terminal amino acid residues that form the “amino terminal Cu(II), Ni(II)” (ATCUN) binding motif, first found in albumin, but bind Cu(II) with lower affinity (Kd in the range of 10^−7^–10^−8^, vs. 10^−11^ or lower) [74]. Histatins are known for their antifungal (as well as healing) properties, and antifungal activity is associated with the N-terminal region of the peptide. Recent studies have shown that activity against yeast (candida albicans) increased 5-fold upon the addition of Cu(II) in the case of histatin-5, EC_50_ concentrations falling from ~5 to ~1 µM [74]. Two His residues near the ATCUN motif were also important for the enhancement of antifungal activity, and other adjacent residues forming a Cu(I) complex may be important. It is noteworthy that the exposure of histatins to saliva, which contains some proteases, did not destroy the antifungal portions, at least initially [76]. However, in saliva, histatin-5 can bind salivary proteins, including amylase, and amylase binding inhibits its antifungal activity [77]. Whether histatins are secreted with Cu attached, however, is unknown. Most salivary Cu is with material less than 5 kDa and thus might be with histatins. Alternatively, traces of Cu ions in water or freed from food during initial digestion in the mouth might bind to the histatins. 

Copper in Gastric, Pancreatic and Duodenal Fluids: Reports on the Cu contents of gastric and duodenal juices per se are hard to find, in part perhaps because it is difficult to obtain “pure” samples not contaminated with secretions from other sources, like saliva in the stomach, and pancreatic and biliary fluids in the upper small intestine. Data compiled by Owen in 1982 (covering reports from the 1950s to the 1980s) [78] gave a value of 0.4 mg/L (~6 µM) for gastric juice [1], and the only more recent one discovered [79] gave a 5-fold lower value (1.2 µM). In the latter case, pains were taken to preclean the gastroscope used for sampling (which was demonstrated to be a source of contamination). Therefore, the lower value may be more reliable. Assuming that an average of 2.5 L of gastric juice are secreted per day [1] at a concentration of 1.2 µM, the daily excretion of Cu through gastric fluid would amount to 0.18 mg of Cu. No reports on the Cu contents of actual duodenal secretions were found, except as a way to determine the contribution of pancreatic fluid, by measuring the increase in Cu in the duodenum in response to secretin [80]. However, anecdotally, in our laboratory we have used enterocytic monolayers of Caco2 cells with tight junctions grown on filters in bicameral chambers and found that radioactively labeled Cu ions administered to the basolateral (“blood” side) rapidly move through the cells and are released at the apical surface, i.e., into the “intestinal lumen” (Luis Corona, Theodros Z. Kidane, Maria C. Linder, unpublished). The nature of this Cu has not been investigated. For pancreatic fluid, there are more determinations, and recent values corroborate those of the past (0.32 versus 0.25–0.35 mg Cu/L) [81,82], which roughly corresponds to 0.4–0.5 mg secreted per day. In all cases, subjects had fasted and were given secretin to stimulate release. Again, very little is known about the nature of the Cu found in these fluids. Cu/Zn SOD (SOD1) was detected in the first portion of pancreatic juice released in response to secretin, but its concentrations fell to very low levels after stimulation with cholecystokinin [11]. All this shows that we still have much to learn about the forms in which Cu is secreted from these various cell types as part of Cu homeostasis. 

## 6. Forms of Cu in Other Fluids and Their Origins

Cu in Urine: In comparison with the amounts of Cu lost from the GI tract on a daily basis (~ 1 mg for the average adult human), those normally lost via the urine are much lower, typically 40–50 µg/day [1,83], but are quite variable: 0.05–0.4 µg/mg creatinine [1,84], and about 12 + 7 µg Cu/L (Mean + SD) in pregnant women near term [85]. Urinary Cu could come from two sources, namely the blood plasma (filtered through the glomerulus) and/or as secretions from certain kidney cells in the tubules. Concerning the latter possibility and as already described, the Cu transporters ATP7A and ATP7B are important mediators of Cu release from cells, whether located in the TGN membrane or trafficking, moving via vesicles to portions of the cell plasma membrane. The protein and mRNA expression of these transporters was examined histologically in different portions of the kidney of normal mice by two different groups [86,87] and yielded partly contradictory results for ATP7A. Grimes et al. [86] found that it was mainly in cells of the proximal and distal tubules, with slight expression in glomerular cells. Moore and Cox [87] found predominant expression in the glomeruli. Either way, expression associated with cells of the nephron raises the possibility that ATP7**A** might be pumping Cu into kidney tubules for excretion (although it normally pumps Cu into the blood). For ATP7**B**, Moore and Cox [87] found it was expressed in the glomeruli as well as in the inner and outer zones of the medulla, presumably associated with the loops of Henle. This localization might support a role for ATP7**B** in secreting Cu into the tubules and thus might also be entering the urine. However, Moore and Cox suggested instead that ATP7B is retrieving Cu from the tubular lumen i.e., is helping to re-absorb it, but this has not been followed up. Further studies by the groups of Lutsenko, Kaplan and Hubbard confirmed the presence of both ATP7A and B in cells of the proximal and distal tubular epithelia [23,88], and discovered that ATP7A but not ATP7B trafficked from the TGN to the cellular periphery near the blood in response to excess Cu [88]. This is consistent with the general concept that ATP7A exports Cu from epithelial cells into the blood and would explain why excess Cu does not accumulate in renal epithelia in Cu overload. (The latter was observed in Atp7b-/- mice.) The reason ATP7B failed to traffic from the TGN to the cell periphery in response to Cu treatment [88] was ascribed to the fact that a modified (2–3 kDa shorter) form was expressed. Indeed, these investigators proposed that in this form, ATP7B might have the role of pumping excess Cu into vesicles for storage. The latter findings suggest that it is unlikely that either of these transporters directly mediate secretion of Cu into the renal tubules for excretion in the urine, although it is clear that follow-up is needed. 

**Figure 6:**
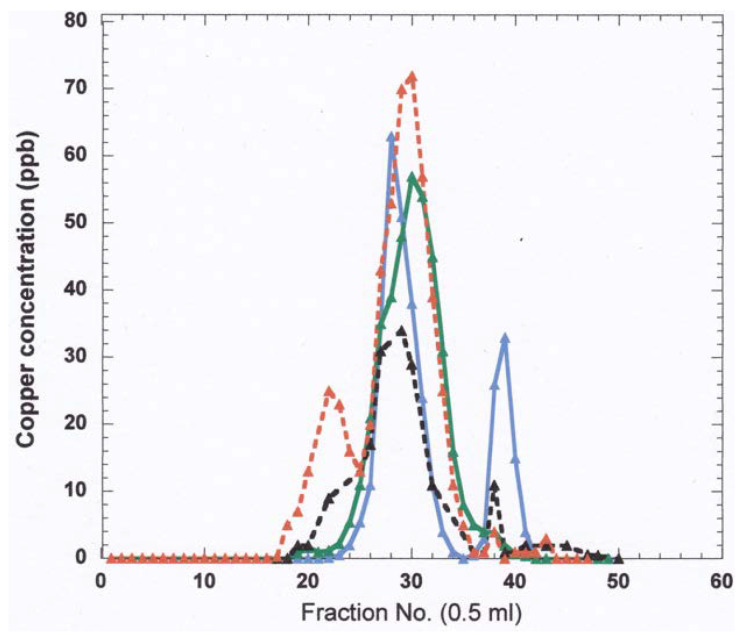
Separation of Cu components of blood plasma from various species showing some Cu eluting at the end of the column volume, where the small Cu carrier (SCC) elutes. Whole plasma samples (100 uL) from humans (red), pigs (blue), sheep (green), and cows (black) were applied to a 25 mL column of Superdex 200, and the Cu content of 0.5 mL fractions collected was measured by furnace atomic absorption spectrometry (Linder et al., unpublished.) The peak of ceruloplasmin elution is in the region of fraction 29–30, and albumin is right after. Alpha-2-macro-globulin elution peaks at about fraction 22.

With regard to blood plasma as a Cu source for the urine, very little Cu is normally associated with components sufficiently small to pass through the glomerular fenestrations, which permit materials up to about 30 kDa to pass, most of which are re-absorbed. Only very small amounts of low molecular weight material binding Cu have been detected in normal plasma [65], and small amounts of Cu are also associated with other components (most likely proteins) smaller than albumin and possibly of a size that might penetrate the glomeruli. However, there normally is very little Cu in the urine, and what little evidence we have suggests it is mainly quite small in size. For example, already in 1981 Suzuki and colleagues [89,90] reported that a major portion of urinary Cu eluted at the end of the column volume in size exclusion chromatography on Sephadex G75. Size exclusion separation and Cu analyses of blood plasma from many species has established that traces of Cu are associated with very small components [67,91]. Figure 6 shows that most of the Cu eluted from whole blood plasma of different species in size exclusion FPLC on Superdex 200 eluted with large proteins, but small and variable amounts eluted with low molecular weight components, centering on fraction 43 (~ 86% of the column volume). In preliminary recent studies of normal human plasma in our own laboratory, individuals had detectable Cu in 3 kDa ultrafiltrates averaging 4.4 + 1.2 ng/mL (mean + SD; N = 4) (Alex Nguyen, Maria C Linder, unpublished data), which one would expect would be filtered by the kidney. Thus, blood fluid of normal mammals can contain very small amounts of one or more low molecular weight Cu-containing components that could be entering the urine. One of the other smaller components in urine may be metallothioneins (SEC Mr ~ 14 kDa; actual mass ~8 kDa), which have been detected there, particularly in the case of copper-loaded rats [12], and can also be present in the bile (see earlier).

**Figure 7:**
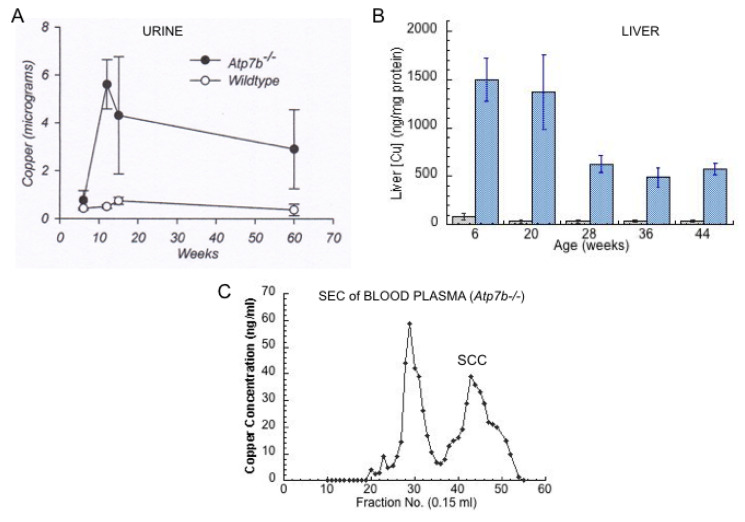
Cu in livers, urine and blood plasma of wild type (Control) and Wilson disease (Atp7b-/-) model mice. Elevations of urinary Cu (**A**) and liver Cu (**B**) (blue vs. grey bars) in the Atp7b-/- mice vs. wild type, and evidence for the presence of large amounts of low molecular weight Cu in the blood plasma (**C**) seen in Superdex 200 size exclusion chromatography (SCC, fractions 40–50). Figure A reprinted with permission, from Gray et al. [93]; data in B replotted from those in Table 1 of Huster et al. [94]; data in C are from Miguel Tellez, Abigael Muchenditsi, Svetlana Lutsenko, and Maria C. Linder, unpublished.

**Figure 8:**
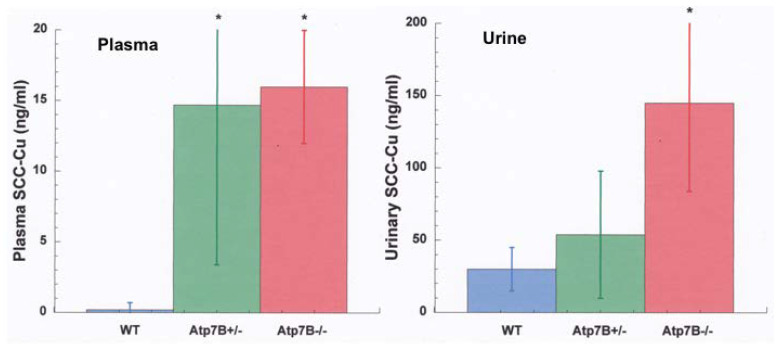
Levels of Cu associated with the small copper carrier (SCC) in the plasma (left) and urine (right) of Labrador retrievers that do and do not have defects in Atp7B, relative to the wild type. Values are for ng/mL Cu in 3 kDa ultrafiltrates (Means + SD) for blood plasma (left) and urine (right) of wild type controls (blue), and for retrievers with hetero (green) and homozygous (red) defects in Atp7b (5–6 animals/group). Pooled ultrafiltrate samples eluted as a single peak of ~1.7 kDa by size exclusion chromatography from a small pore gel column (Superdex 30 Increase) (not shown). From Sai Vallabhaneni, Kaitlynne Kim, Maria C. Linder and Hille Fieten, unpublished. * *p* < 0.01 vs. WT.

Studies of Cu overload are adding to our understanding of urinary Cu. When there is overload, as in Wilson disease (WD), as well as in animals fed high Cu diets or injected with large amounts of Cu, urinary Cu levels rise dramatically. For example, in rats on a high Cu diet, urinary Cu increased 2.5-fold in 5 weeks, and 4-fold by 16 weeks (liver MT increased 3.5–5-fold) [12]. In human WD subjects, Walshe reported that pre-symptomatic patients excreted an average of 208 µg Cu/24 h [92], compared with about 40 µg in normal subjects [83]. WD patients with full blown hepatic and neurological WD had even higher average levels, excreting an average of 466 and 306 µg/day in the urine, respectively [92]. Figure 7A shows data from Gray et al. [92] on the elution of large amounts of urinary Cu by WD model mice, compared to controls, in conjunction with accumulation of large amounts of Cu in the liver (Figure 7B). In this case, a large increase in the levels of low-molecular weight Cu in the blood plasma was also observed [67] (Figure 7C). This would be consistent with the latter being responsible for the former, i.e., low-molecular weight Cu in the plasma was filtering into the urine. We obtained further preliminary results on 3 kDa ultrafiltrates of plasma and urine from Labrador retrievers without and with ATP7B mutations and Cu overload, showing that a lack of ATP7B function markedly increased levels of low-molecular weight Cu in both fluids, relative to the norm (wild type) (Figure 8). Thus, blood fluid rather than kidney cells is most probably the main source of the increased low-molecular weight Cu excreted in the urine of Cu overloaded animals, and the same is likely to be true in humans.

These various findings imply that under normal conditions, the very low levels of Cu in urine are associated with mostly unknown components, but might include MT and possibly other minor substances secreted into urinary tubules by kidney epithelial cells, as well as traces of a low molecular weight component also found in the blood fluid. However, when there are large increases either in the intake/absorption of Cu from the diet and/or genetic changes that cause liver Cu accumulation, urinary Cu excretion can markedly expand, and most of the additional Cu entering the urine probably comes from low molecular weight Cu in the blood plasma. The nature of the main components, termed the small copper carrier (SCC), appearing in the urine in Cu overload, was initially investigated by Gray et al. [93] using size exclusion and anion exchange chromatography, with the outcome that it was 1–2 kDa, and had a negative charge at neutral pH. Further characterization of this SCC is in progress, mainly in our laboratory, and confirms it is an anion (by native PAGE and isoelectric focusing), may have a mass to charge ratio of 845 (determined by electrospray MS), and contains Cu^2+^ most likely bound to N and O ligands (determined by EPR) [Bertrand Vileno and Maria Linder et al., unpublished]. Purification of sufficient amounts of this component for structure determination is proceeding. The small Cu carrier is secreted by several cultured cell types, and thus may have an important role not only to reduce excessive copper loads in the liver and other organs by enhancing its urinary excretion [92] but also in the distribution of Cu to other tissues via the circulation, all of which are related to mammalian Cu homeostasis. Whether (and, if so, how) this SCC may relate to the “free Cu” or “non-ceruloplasmin Cu” in serum that has been measured by a patented procedure and may relate to Alzheimer’s disease [95] remains to be determined.

## 7. Conclusions

**Figure 9:**
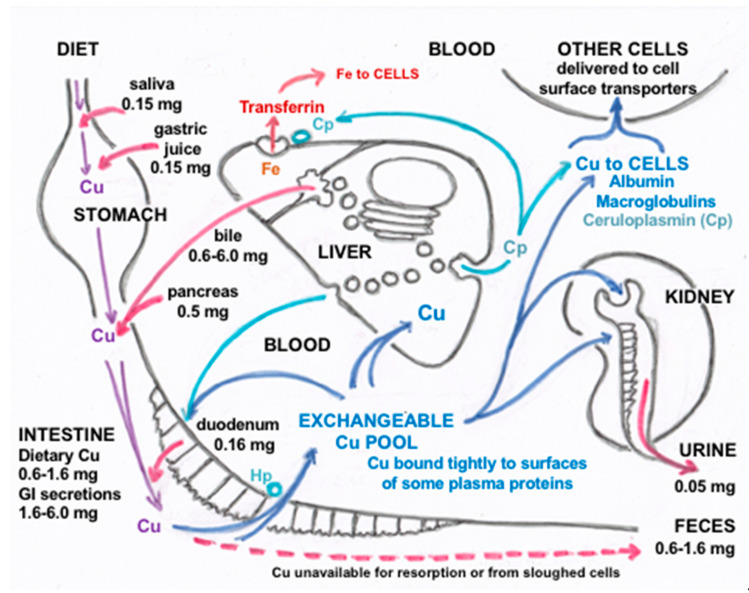
Overview and Summary of the homeostatic processes described in this review for the normal human adult. This figure diagrams amounts of copper entering the digestive tract from various sources and how they travel from the intestine to key organs (like liver and kidney) and the rest of the organism, as well as how copper is excreted. Values indicated are average mg Cu per day. Dietary Cu is indicated by purple lines; absorption and distribution to liver, kidney and other cells is in darker blue; involvement of ceruloplasmin (Cp) is in lighter blue; involvement of Cp and hephaestin (Hp) in Fe efflux from liver and intestine, respectively, is in the light blue and orange; secretions that lead to Cu efflux into the GI tract, feces and urine are in red. Modified and updated from Linder [9].

The main and best established points about how mammalian Cu homeostasis occurs through a balance between absorption, transport and excretion, and the important roles of secretions discussed in this review are summarized in Figure 9. The figure depicts the amounts of Cu entering the digestive tract from various sources and how they travel from the intestine to key organs (like liver and kidney) and the rest of the organism, as well as how Cu is excreted. Values indicated are average mg Cu per day, based on the current and earlier literature, as previously discussed. Starting with Cu in food and drink entering the mouth (top left; “Diet”), as well as that in salivary, gastric, biliary and pancreatic secretions, plus some Cu from the intestinal mucosal cells of the small intestine, provide Cu to the GI tract. Most of that is absorbed in the duodenum of the small intestine.

Transfer of absorbed Cu from the intestinal cells to the blood is accomplished mainly by the Cu ATPase (ATP7A). Based upon tracer studies with radioactive Cu, Cu that enters the hepatic portal circulation binds tightly to proteins that compose the exchangeable Cu pool of the blood plasma, which consists mainly of albumin and alpha-2-macroglobulin (transcuprein), but may include other protein or non-protein components. The bulk of this Cu then first enters cells of the liver and partly also those of the kidney (Figure 2), where Cu is supplied to endogenous Cu-dependent proteins via various chaperones (ATOX1, CCS, Cox17). Most travels to the transmembrane Cu “pump”, ATP7B, in the trans Golgi network, from where a significant portion is incorporated into ceruloplasmin (Cp) for secretion into the blood via exocytosis, where it joins Cu on albumin and transcuprein/macroglobulin for delivery to cells in other organs all over the body (including the intestine). Another portion of Cp interacts with Fe (orange section of the diagram) to facilitate Fe release and transport on its blood carrier, transferrin. Hephaestin (Hp), a membrane-bound homolog of Cp, plays a similar role in Fe release from enterocytes to the blood. Excess Cu in the liver cells finds its way into the bile after entering the bile canaliculi, with the help of ATP7B and the endo-lysosomal compartment (see Figure 4). Most excretion of Cu is via the bile, as biliary Cu is not as readily re-absorbed, and so ends up in the feces. Cells regularly sloughed off the intestinal mucosa and degraded in the large intestine may also contribute to fecal excretion. Normally, only a small amount of Cu is excreted in the urine, which probably derives not only from tubular secretions but also the filtration of blood plasma. However, urinary secretion markedly increases when biliary secretion is compromised, leading to increases in small Cu carriers (SCC) in blood plasma that greatly increase levels of SCC in the urine. A great deal is still unknown about the additional forms of Cu that may be involved in its flow, especially through various secretory pathways leading to the blood, bile and urine, and the contributions of these processes and components to mammalian Cu homeostasis, as described in this review.

## Figures and Tables

**Table 1:** Copper in Fluids of the Human Gastrointestinal Tract and Urine *.

Fluid	[Cu] mg/L	(uM)	Volume (L/day)	Total Cu (mg)
Saliva	0.1	1.6	1–2	0.15
Gastric juice	0.08	1.2	2.5	0.18
Pancreatic juice	0.3	4	1.5	0.5
Bile	4 ± 2	19–63	0.5–0.75	0.6–6
Duodenal fluid	0.016		(1?)	0.016
Urine	(0.02–0.1) **	(0.03–0.10)	1.5	0.04–0.05 ***

* Updated from Table 1-5, Biochemistry of Copper, Linder, 1991 [1]; ** calculated based on the Total Cu values and average urinary volume; *** solid values obtained in past and recent studies [11,12,13].

**Table 2:** Absorption and Dialysability of Cu in GI Tract Fluids of Rats *.

Fluid	Dialysable Cu (Percent ^64^Cu)	Intestinal Absorption (Percent)	Body retention (Percent ^64^Cu)
In vitro ^64^Cu labeled ^a^			
Saliva	98 ± 2 (6)		13 ± 3 (6)
Gastric juice	97 ± 2 (6)		12 ± 10 (6)
Bile (gall bladder)	12 ± 12 (5)		6 ± 1 (6)
In vivo ^64^Cu-labeled ^b^			
Time after intubation			
Bile 0–4 h	50 ± 7 (9)	16 ± 3 (8)	
4–8 h	28 ± 6 (9)	13 ± 4 (8)	
8–24 h	16 ± 3 (9)	10 ± 3 (4)	

* Summarized from Biochemistry of Copper, Linder, 1991 [1]. Means ± SD (N). ^a^ Table 5-3, ^b^ Table 5-2.

**Table 3:** Tabulated data on rates of ^67^Cu loss in rats where the common bile duct was and was not (sham) ligated prior to ^67^Cu administration. Reprinted with permission from Linder, M.C. et al. [2].

	Sham-Operated Controls	Bile Duct Ligation
Slope [Means ± SD (N)] * 0–63 h 0–86 h	−552 ± 28 (23) −623 ± 56 (28)	−258 ± 97 (15) ** −272 ± 67 (17) ***
Half-life (h) 0–63 h 0–86 h	54 h 49 h	117 h 110 h
Liver [Means ± SD (N)] Cu (ug/g) ^67^Cu specific activity (cpm/ug)	4.6 ± 0.5 (6) 64 ± 11 (4)	3.8 ± 1.1 (6) 105 ± 42 (5)

* Multiplied by 10^6^. ** *p* < 0.01, *** *p* < 0.001.
